# Grip strength in the association between frailty and new-onset depression in older adults: a multinational longitudinal study

**DOI:** 10.3389/fpubh.2026.1810444

**Published:** 2026-06-03

**Authors:** Lijian Han, Yuanying Song, Rong Luo, Shufang Wang, Yuan Shen, Gendi Wang

**Affiliations:** 1 Department of Neurology, Yancheng Third People's Hospital (Affiliated Hospital 6 of Nantong University, The Yancheng School of Clinical Medicine of Nanjing Medical University, The affiliated hospital of Jiangsu Vocational College of Medicine), Yancheng, Jiangsu, China; 2 School of Pharmacy, Nanjing Medical University, Nanjing, Jiangsu, China

**Keywords:** competing risk, frailty index, mediation effect, muscle strength, new-onset depression, older adults

## Abstract

**Background:**

Frailty is a multidimensional geriatric syndrome characterized by cumulative physiological decline. While associated with various adverse outcomes, the longitudinal relationship between frailty and new-onset depression across diverse national populations remains insufficiently explored. This study investigated the association between the Frailty Index (FI) and new-onset depression in three large aging cohorts.

**Methods:**

This multinational longitudinal cohort study included community-dwelling adults aged ≥50 years from three nationally representative aging cohorts: the Health and Retirement Study (HRS), the English Longitudinal Study of Aging (ELSA), and the Survey of Health, Aging and Retirement in Europe (SHARE). Frailty was assessed using a deficit accumulation–based frailty index (FI). New-onset depression was defined by validated CES-D 8 or EURO-D scores among participants free of depression at baseline. Associations were estimated using Cox proportional hazards, Fine–Gray competing risk, and discrete-time logistic regression models. Dose–response patterns were examined using restricted cubic splines, and mediation analysis evaluated the role of grip strength.

**Results:**

Over median follow-ups of 8 (HRS), 2 (ELSA), and 4 (SHARE) years, 749, 973, and 7,821 participants developed new-onset depression, respectively. Cohort-specific incidence rates were 34.5% in HRS (749/2, 171), 18.6% in ELSA (973/5, 223), and 30.4% in SHARE (7, 821/25, 721). Adjusted analyses showed the FI was consistently associated with depression risk across all cohorts. Each one-unit FI increase was associated with an approximately 4% higher risk (HR = 1.04, *p* < 0.001 for all cohorts). Participants in the highest FI quartile had significantly elevated risk compared to the lowest. Results were robust across all statistical models. Restricted cubic splines indicated a nonlinear association, which was partially mediated by grip strength.

**Conclusion:**

Frailty is an independent predictor of new-onset depression in older adults across diverse international populations. Early frailty identification and targeted multidimensional interventions—including grip strength enhancement, structured physical activity, nutritional optimization, and psychosocial support—may help reduce depression incidence and promote healthy aging.

## Introduction

Global populations are aging rapidly. The proportion of individuals aged 65 years and older is projected to rise from approximately 10% in 2022 to 16% by 2050 (1). This trend is especially pronounced in high-income countries, where life expectancy has increased substantially. By mid-century, the global population aged 60 years and older is expected to reach 2.1 billion (2). In parallel, depression has become a major public health concern among older adults, with prevalence estimates ranging from 19.2 to 49.9% across populations (3, 4). Depression accounts for more than 10% of disability-adjusted life years (DALYs) among individuals aged 60 years and older and contributes substantially to the global burden of disease (5). Late-life depression is associated with accelerated functional decline, increased mortality, and greater healthcare utilization, including longer hospital stays and higher medical expenditures (6, 7). In this context, new-onset depression refers to incident depressive symptoms or depressive disorder occurring during follow-up among individuals without baseline depression, thereby providing stronger temporal evidence than cross-sectional prevalence estimates and enabling evaluation of dynamic risk trajectories.

Frailty is defined as a state of diminished physiological reserve and heightened vulnerability to stressors, resulting from cumulative declines across multiple organ systems (8). Two major conceptual models have been proposed: the Fried phenotype and the frailty index (FI). The Fried phenotype defines frailty based on five criteria—unintentional weight loss, exhaustion, weakness, slow gait speed, and low physical activity—whereas the FI quantifies accumulated health deficits across multiple domains (9). As a continuous measure, the FI is more sensitive to gradations in health status and facilitates comparisons across populations (10). Frailty, particularly when assessed using the FI, has been strongly linked to adverse outcomes, including mortality, disability, and hospitalization (11). Muscle strength, most commonly operationalized as grip strength in epidemiological studies, reflects overall muscular function and physiological reserve and is widely recognized as an accessible biomarker of sarcopenia, frailty progression, and functional aging.

A growing body of literature has examined the frailty–depression relationship. Systematic reviews and meta-analyses have consistently shown strong associations between frailty and depressive symptoms, but much of this evidence is based predominantly on cross-sectional studies, limiting causal and temporal inference (12, 13). Several longitudinal investigations, including studies conducted in HRS-, ELSA-, or SHARE-related populations, have further suggested prospective links between frailty and subsequent depression; however, many were restricted by single-country analyses, heterogeneous frailty definitions, limited cross-cohort harmonization, or narrower analytical frameworks (14–16). Importantly, although these datasets have been individually used to study aging-related mental health outcomes, few studies have simultaneously integrated HRS, ELSA, and SHARE to systematically evaluate frailty–depression associations across multiple healthcare systems and sociocultural contexts using harmonized FI methodology (17).

Most studies investigating the association between frailty and depression in older adults are cross-sectional, limiting conclusions about temporal sequence and causality (18). Existing longitudinal studies are relatively few and often constrained by small sample sizes or single-country designs, thereby restricting generalizability (19). Moreover, prior studies rarely incorporated competing-risk frameworks to account for death, systematically assessed nonlinear dose–response patterns, or examined grip strength as a potential intermediary mechanism linking frailty to incident depression. Large multinational longitudinal studies are needed to validate the robustness of the association between frailty and new-onset depression and to clarify potential mechanisms.

Frailty and depression may share common biological pathways. Chronic inflammation, characterized by elevated cytokine levels, has been implicated in both conditions (20). Neuroendocrine dysregulation, particularly dysfunction of the hypothalamic–pituitary–adrenal axis, may further increase vulnerability by disrupting cortisol regulation and exacerbating depressive symptoms (21). Sarcopenia and muscle weakness may serve as intermediate mechanisms, as reduced physical capacity contributes to functional decline and social isolation (22). Social vulnerability and reduced engagement may reinforce this cycle. Emerging evidence suggests that grip strength may act as a key mediator linking frailty to depression.

Using data from HRS, ELSA, and SHARE, the present study aimed to evaluate the longitudinal association between the frailty index and new-onset depression, assess death as a competing risk, examine potential dose–response relationships, and investigate the mediating role of grip strength.

## Materials and methods

### Study population

The present longitudinal cohort study was based on data from three nationally representative aging cohorts: the Health and Retirement Study (HRS, United States), the English Longitudinal Study of Aging (ELSA, England), and the Survey of Health, Aging and Retirement in Europe (SHARE, multiple European countries). These cohorts were selected because they employ broadly comparable longitudinal designs, biennial follow-up frameworks, and standardized assessments of health, functional status, socioeconomic conditions, and physical performance among adults aged ≥50 years, thereby enabling harmonized cross-national analyses.

HRS, ELSA, and SHARE were specifically chosen due to their strong methodological alignment and the availability of sufficiently comparable variables required for standardized frailty index construction, new-onset depression assessment, and grip strength evaluation. This harmonization allowed robust examination of frailty–depression associations across diverse healthcare systems and sociocultural settings while minimizing methodological heterogeneity.

Although other aging cohorts, such as the China Health and Retirement Longitudinal Study (CHARLS), also provide valuable longitudinal data, they were not included because differences in measurement frameworks, variable harmonization, and cross-cohort comparability could substantially increase heterogeneity and compromise direct multinational comparability within the present analytical framework. Therefore, our study prioritized methodological consistency across highly harmonized cohorts to strengthen internal validity and cross-national interpretability.

**Figure 1:**
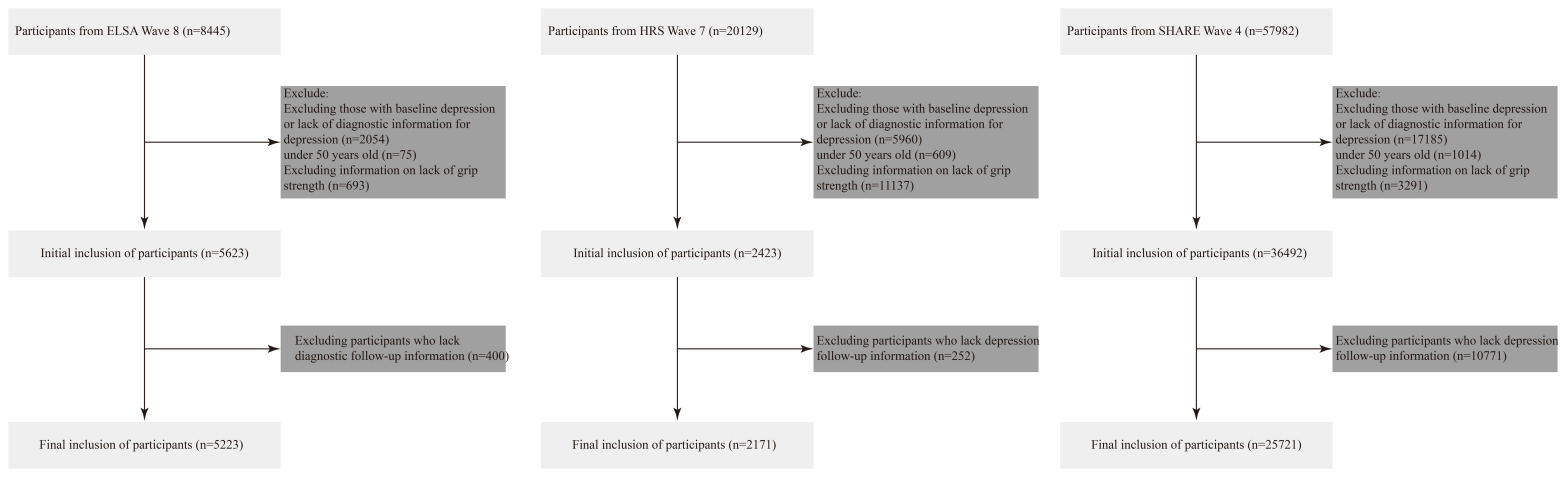
Flowcharts of participant selection in the HRS, ELSA, and SHARE cohorts.

Participants were selected from ELSA, HRS, and SHARE according to predefined inclusion and exclusion criteria, including age eligibility, absence of baseline depression, availability of grip strength measurements, and complete follow-up data for incident depression assessment. After sample selection, the final analytical populations included 5,223 participants from ELSA, 2,171 from HRS, and 25,721 from SHARE. Detailed participant selection procedures and exclusion criteria for each cohort are presented in Figure 1.

### Assessment of the frailty index

The frailty index (FI) was constructed using the deficit accumulation approach based on the framework proposed by Rockwood and Mitnitski. Health deficits were selected according to established criteria: each variable required clear biological relevance, a documented association with adverse health outcomes, an increasing prevalence with age, and no early saturation in the general older population. The selected deficits encompassed multiple domains, including chronic diseases, functional limitations, activities of daily living, self-rated health, cognitive function, and psychological status.

All deficit variables were coded on a scale from 0 to 1, where 0 indicated absence and 1 indicated full presence of the deficit. Ordinal variables were assigned proportional scores (e.g., 0, 0.5, 1) according to severity. For each participant, the FI was calculated by dividing the number of present deficits by the total number of deficits considered. To ensure the stability of FI estimation, individuals with more than 20% missing deficit items were excluded.

In the primary analyses, the FI was modeled as a continuous variable. For descriptive and survival analyses, the FI was additionally categorized into cohort-specific quartiles. Higher FI values indicate greater frailty severity.

### Assessment of new-onset depression

Depressive symptoms were assessed using validated instruments in each cohort. In HRS and ELSA, the 8-item Center for Epidemiologic Studies Depression Scale (CES-D 8) was administered. In SHARE, depressive symptoms were measured using the 12-item EURO-D scale. Both instruments have been extensively validated in older populations and demonstrate adequate reliability and validity.

Only participants without depressive symptoms at baseline were included in the longitudinal analyses. New-onset depression was defined as the first occurrence of depressive symptoms during follow-up. In HRS and ELSA, a CES-D 8 score ≥3 indicated depression. In SHARE, a EURO-D score ≥4 was used. These thresholds were based on commonly applied cutoffs in previous studies.

Follow-up time was calculated from baseline to the first onset of depressive symptoms or the last available follow-up assessment, whichever occurred first.

### Covariates

Sociodemographic characteristics and lifestyle factors were included as covariates. These variables comprised age, sex, race, education, wealth, body mass index (BMI), drinking status, smoking status, and physical activity. Smoking status was categorized as never smoker or current smoker. Drinking status was classified as non-drinker or current drinker. BMI was calculated as weight (kg) divided by height squared (m^2^).

### Statistical analyses

Continuous variables were summarized as means and standard deviations (SD), and categorical variables were presented as counts and percentages. Between-group differences were assessed using chi-square tests or Student's *t* tests, as appropriate.

Multivariable Cox proportional hazards regression models were used to examine the association between FI and new-onset depression. Time to event was defined as the interval from baseline to the first occurrence of depression, censoring, or end of follow-up. Hazard ratios (HRs) and 95% confidence intervals (CIs) were estimated. Four models were specified: Model 1 was unadjusted; Model 2 adjusted for age, sex, race, education, wealth, and BMI; Model 3 additionally adjusted for drinking and smoking status; and Model 4 further adjusted for physical activity. The proportional hazards assumption was evaluated using Schoenfeld residuals, and no substantial violations were observed.

Given that death may preclude the occurrence of depression, competing risk analyses were conducted using the Fine–Gray subdistribution hazards model, with death treated as a competing event. Subdistribution hazard ratios (sHRs) and 95% CIs were calculated to assess the robustness of the findings.

To evaluate robustness under an alternative time specification, discrete-time logistic regression models were fitted using follow-up waves as the time unit. The dataset was restructured into a person-period format, in which each participant contributed one observation per wave until new-onset depression or censoring.

Restricted cubic spline analyses were performed to assess potential nonlinear associations between FI and new-onset depression. Subgroup analyses and interaction tests were conducted to explore potential effect modification.

Mediation analysis was conducted to examine the potential mediating role of the frailty index in the association between baseline cardiovascular disease and incident depressive disorder. In each cohort, the FI was standardized to facilitate comparison of effect estimates between HRS and ELSA.

All statistical analyses were performed using IBM SPSS Statistics (version 24.0) and R software (version 4.3.0). Two-sided *p* values < 0.05 were considered statistically significant.

Data extraction, harmonization, and preprocessing were performed using standardized protocols by trained investigators with experience in epidemiological and longitudinal cohort data analysis. Key variables, including frailty index components, depression outcomes, grip strength measures, and covariates, were independently extracted and cleaned according to predefined criteria. To ensure data accuracy and cross-cohort consistency, cleaned datasets and coding procedures were cross-verified by at least two researchers, with discrepancies resolved through repeated review and consensus. This quality-control process was implemented to minimize data-entry errors, improve harmonization reliability, and strengthen reproducibility across HRS, ELSA, and SHARE datasets.

## Results

### Baseline characteristics according to new-onset depression status

Tables 1–3 summarize baseline characteristics across HRS, ELSA, and SHARE. Across all three cohorts, participants who developed new-onset depression during follow-up consistently exhibited a less favorable baseline health profile than those who remained depression-free. Specifically, incident depression was more common among women, individuals with lower socioeconomic status, higher body mass index, lower grip strength, and substantially higher frailty index (FI) values. Despite differences in follow-up duration and demographic composition across cohorts, these patterns were remarkably consistent, suggesting a robust cross-national relationship between baseline frailty-related vulnerability and future depression risk.

**Table 1:** Baseline characteristics of participants according to new-onset depression status in the HRS cohort.

Characteristic	*N*	Overall *N* = 2,171	Normal *N* = 1,422	New-onset depression *N* = 749	*p*-value
Sex^b^, *n* (%)					
Female	2,171	1,247 (57.44)	757 (53.23)	490 (65.42)	< 0.001
Male		924 (42.56)	665 (46.77)	259 (34.58)	
Age (year)^a^, mean ± SD	2,171	69.11 ± 11.25	69.25 ± 11.27	68.83 ± 11.22	0.409
Race^b^, *n* (%)					
Non-Hispanic white	2,171	1,795 (82.68)	1,218 (85.65)	577 (77.04)	< 0.001
Non-Hispanic blacks		182 (8.38)	100 (7.03)	82 (10.95)	
Hispanic		151 (6.96)	81 (5.70)	70 (9.35)	
Other race		43 (1.98)	23 (1.62)	20 (2.67)	
Education^b^, *n* (%)					
High school or less	2,171	439 (20.22)	239 (16.81)	200 (26.70)	< 0.001
Some college		1,203 (55.41)	794 (55.84)	409 (54.61)	
College graduate or above		529 (24.37)	389 (27.36)	140 (18.69)	
Wealth^a^, Mean ± SD	2,171	479,559.48 ± 951,933.53	521,837.82 ± 1,049,339.93	399,292.72 ± 726,093.36	< 0.001
BMI^a^, Mean ± SD	2,171	27.21 ± 5.22	27.02 ± 5.06	27.57 ± 5.51	0.024
Smoking status^b^, *n* (%)					
No	2,171	1,003 (46.20)	668 (46.98)	335 (44.73)	0.318
Yes		1,168 (53.80)	754 (53.02)	414 (55.27)	
Drinking status^b^, *n* (%)					
No	2,171	1,041 (47.95)	651 (45.78)	390 (52.07)	0.005
Yes		1,130 (52.05)	771 (54.22)	359 (47.93)	
Physical activity^b^, *n* (%)					
No	2,171	725 (33.39)	447 (31.43)	278 (37.12)	0.008
Yes		1,446 (66.61)	975 (68.57)	471 (62.88)	
Grip strength^a^, Mean ± SD	2,171	29.60 ± 11.30	30.44 ± 11.61	28.01 ± 10.50	< 0.001
Frailty^a^, mean ± SD	2,171	16.74 ± 10.54	15.44 ± 10.22	19.20 ± 10.70	< 0.001
Frailty group^b^, *n* (%)					
Q1	2,171	542 (24.97)	406 (28.55)	136 (18.16)	< 0.001
Q2		542 (24.97)	382 (26.86)	160 (21.36)	
Q3		544 (25.06)	341 (23.98)	203 (27.10)	
Q4		543 (25.01)	293 (20.60)	250 (33.38)	

^a^Student *t*-test, ^b^Chi-square test, SD, standard deviation. Some college: attended college or post-secondary education beyond high school without completion of a bachelor's degree.

**Table 2:** Baseline characteristics of participants according to new-onset depression status in the ELSA cohort.

Characteristic	N	Overall *N* = 5,223	Normal *N* = 4,250	New-onset depression *N* = 973	*p*-value
Sex^b^, *n* (%)					
Female	5,223	2,917 (55.85)	2,267 (53.34)	650 (66.80)	< 0.001
Male		2,306 (44.15)	1,983 (46.66)	323 (33.20)	
Age (year)^a^, mean ± SD	5,223	69.25 ± 8.56	69.09 ± 8.30	69.93 ± 9.57	0.029
Race^b^, *n* (%)					
No white	5,223	151 (2.89)	111 (2.61)	40 (4.11)	0.012
White		5,072 (97.11)	4,139 (97.39)	933 (95.89)	
Education^b^, *n* (%)					
Before high school	5,223	1,813 (34.71)	1,371 (32.26)	442 (45.43)	< 0.001
High school		1,126 (21.56)	922 (21.69)	204 (20.97)	
Junior college		1,245 (23.84)	1,037 (24.40)	208 (21.38)	
College graduate or above		1,039 (19.89)	920 (21.65)	119 (12.23)	
Wealth^a^, mean ± SD	5,223	468,346.74 ± 847,923.98	505,914.04 ± 914,787.00	304,255.23 ± 413,976.24	< 0.001
BMI^a^, mean ± SD	5,223	28.09 ± 5.17	27.87 ± 4.98	29.01 ± 5.84	< 0.001
Smoking status^b^, *n* (%)					
No	5,223	2,000 (38.29)	1,647 (38.75)	353 (36.28)	0.152
Yes		3,223 (61.71)	2,603 (61.25)	620 (63.72)	
Drinking status^b^, *n* (%)					
No	5,223	614 (11.76)	428 (10.07)	186 (19.12)	< 0.001
Yes		4,609 (88.24)	3,822 (89.93)	787 (80.88)	
Physical activity^b^, *n* (%)					
No	5,223	797 (15.26)	485 (11.41)	312 (32.07)	< 0.001
Yes		4,426 (84.74)	3,765 (88.59)	661 (67.93)	
Grip strength^a^, mean ± SD	5,223	28.13 ± 10.52	28.97 ± 10.38	24.49 ± 10.37	< 0.001
Frailty^a^, mean ± SD	5,223	16.79 ± 12.81	14.49 ± 10.74	26.80 ± 15.96	< 0.001
Frailty group^b^, *n* (%)					
Q1	5,223	1,285 (24.60)	1,212 (28.52)	73 (7.50)	< 0.001
Q2		1,325 (25.37)	1,180 (27.76)	145 (14.90)	
Q3		1,303 (24.95)	1,071 (25.20)	232 (23.84)	
Q4		1,310 (25.08)	787 (18.52)	523 (53.75)	

^a^Student *t*-test, ^b^Chi-square test, SD, standard deviation.

**Table 3:** Baseline characteristics of participants according to new-onset depression status in the SHARE cohort.

Characteristic	N	Overall *N* = 25,721	Normal *N* = 17,900	New-onset depression *N* = 7,821	*p*-value
Sex^b^, *n* (%)					
Female	25,721	13,281 (51.63)	8,443 (47.17)	4,838 (61.86)	< 0.001
Male		12,440 (48.37)	9,457 (52.83)	2,983 (38.14)	
Age (year)^a^, mean ± SD	25,721	65.19 ± 9.29	64.50 ± 8.99	66.77 ± 9.78	< 0.001
Education^b^, *n* (%)					
Before high school	25,721	9,325 (36.25)	5,878 (32.84)	3,447 (44.07)	< 0.001
High school and vocational training		10,238 (39.80)	7,366 (41.15)	2,872 (36.72)	
Higher education		6,158 (23.94)	4,656 (26.01)	1,502 (19.20)	
Wealth ^a^, mean ± SD	25,721	325,339.76 ± 600,766.30	346,410.04 ± 635,775.44	277,115.99 ± 508,466.59	< 0.001
BMI ^a^, mean ± SD	25,721	26.77 ± 4.43	26.65 ± 4.35	27.03 ± 4.61	< 0.001
Smoking status^b^, *n* (%)					
No	25,721	13,485 (52.43)	9,218 (51.50)	4,267 (54.56)	< 0.001
Yes		12,236 (47.57)	8,682 (48.50)	3,554 (45.44)	
Drinking status^b^, *n* (%)					
No	25,721	1,917 (7.45)	1,168 (6.53)	749 (9.58)	< 0.001
Yes		23,804 (92.55)	16,732 (93.47)	7,072 (90.42)	
Physical activity^b^, *n* (%)					
No	25,721	1,600 (6.22)	838 (4.68)	762 (9.74)	< 0.001
Yes		24,121 (93.78)	17,062 (95.32)	7,059 (90.26)	
Grip strength^a^, mean ± SD	25,721	33.64 ± 11.42	35.09 ± 11.43	30.34 ± 10.67	
Frailty^a^, mean ± SD	25,721	11.80 ± 8.67	10.49 ± 7.69	14.79 ± 9.96	< 0.001
Frailty group^b^, *n* (%)					
Q1	25,721	6,228 (24.21)	5,048 (28.20)	1,180 (15.09)	< 0.001
Q2		6,660 (25.89)	5,039 (28.15)	1,621 (20.73)	
Q3		6,390 (24.84)	4,345 (24.27)	2,045 (26.15)	
Q4		6,443 (25.05)	3,468 (19.37)	2,975 (38.04)	

^a^Student *t*-test, b, Chi-square test, SD, standard deviation.

### Association between the frailty index and new-onset depression

Across all cohorts, higher FI was consistently associated with increased risk of new-onset depression in both continuous and categorical analyses. This association remained stable after comprehensive multivariable adjustment, indicating that frailty independently predicted depression beyond major sociodemographic, behavioral, and health-related confounders. Each incremental increase in FI was associated with a higher depression risk, while participants in the highest FI quartile experienced substantially greater risk than those in the lowest quartile. Notably, the graded increase across FI quartiles demonstrated a clear dose–response pattern, reinforcing frailty severity as an important longitudinal predictor of depression (Table 4).

**Table 4:** Associations between the Frailty Index and the risk of new-onset depression in the HRS, ELSA, and SHARE cohorts using Cox proportional hazards models.

Exposure variable	Model 1		Model 2		Model 3		Model 4	
	HR (95% CI)	*P*-value	HR (95% CI)	*P*-value	HR (95% CI)	*P*-value	HR (95% CI)	*P*-value
HRS								
15.6-7.4,-14.3498ptFrailty	1.04 (1.04 ~ 1.05)	< 0.001^***^	1.04 (1.03 ~ 1.04)	< 0.001^***^	1.04 (1.03 ~ 1.04)	< 0.001^***^	1.04 (1.03 ~ 1.04)	< 0.001^***^
Frailty group								
Q1	1.00 (reference)	–	1.00 (reference)	–	1.00 (reference)	–	1.00 (reference)	
Q2	1.38 (1.10 ~ 1.74)	0.005^**^	1.33 (1.05 ~ 1.68)	0.017^*^	1.32 (1.04 ~ 1.67)	0.021^*^	1.32 (1.04 ~ 1.67)	0.021^*^
Q3	2.01 (1.62 ~ 2.50)	< 0.001^***^	1.89 (1.50 ~ 2.39)	< 0.001^***^	1.87 (1.48 ~ 2.37)	< 0.001^***^	1.87 (1.48 ~ 2.36)	< 0.001^***^
Q4	3.22 (2.61 ~ 3.99)	< 0.001^***^	2.85 (2.25 ~ 3.60)	< 0.001^***^	2.79 (2.20 ~ 3.53)	< 0.001^***^	2.75 (2.17 ~ 3.49)	< 0.001^***^
15.6-7.4,-14.3498ptP for trend	1.05 (1.04 ~ 1.06)	< 0.001^***^	1.05 (1.04 ~ 1.05)	< 0.001^***^	1.04 (1.03 ~ 1.05)	< 0.001^***^	1.04 (1.03 ~ 1.05)	< 0.001^***^
ELSA								
15.6-7.4,-14.3498ptFrailty	1.05 (1.05 ~ 1.05)	< 0.001^***^	1.05 (1.04 ~ 1.05)	< 0.001^***^	1.05 (1.04 ~ 1.05)	< 0.001^***^	1.04 (1.04 ~ 1.05)	< 0.001^***^
Frailty group								
Q1	1.00 (Reference)	–	1.00 (Reference)	–	1.00 (Reference)	–	1.00 (Reference)	–
Q2	1.98 (1.50 ~ 2.63)	< 0.001^***^	2.04 (1.54 ~ 2.71)	< 0.001^***^	2.05 (1.55 ~ 2.73)	< 0.001^***^	2.08 (1.57 ~ 2.76)	< 0.001^***^
Q3	3.36 (2.58 ~ 4.37)	< 0.001^***^	3.42 (2.61 ~ 4.47)	< 0.001^***^	3.46 (2.64 ~ 4.53)	< 0.001^***^	3.45 (2.63 ~ 4.52)	< 0.001^***^
Q4	8.64 (6.76 ~ 11.03)	< 0.001^***^	8.46 (6.52 ~ 10.98)	< 0.001^***^	8.47 (6.51 ~ 11.03)	< 0.001^***^	7.63 (5.84 ~ 9.97)	< 0.001^***^
15.6-7.4,-14.3498ptP for trend	1.07 (1.07 ~ 1.08)	< 0.001^***^	1.07 (1.06 ~ 1.08)	< 0.001^***^	1.07 (1.06 ~ 1.08)	< 0.001^***^	1.07 (1.06 ~ 1.07)	< 0.001^***^
SHARE								
15.6-7.4,-14.3498ptFrailty	1.04 (1.04 ~ 1.05)	< 0.001^***^	1.04 (1.04 ~ 1.04)	< 0.001^***^	1.04 (1.04 ~ 1.04)	< 0.001^***^	1.04 (1.03 ~ 1.04)	< 0.001^***^
Frailty group								
Q1	1.00 (Reference)	–	1.00 (Reference)	–	1.00 (Reference)	–	1.00 (Reference)	–
Q2	1.32 (1.22 ~ 1.42)	< 0.001^***^	1.28 (1.19 ~ 1.38)	< 0.001^***^	1.27 (1.18 ~ 1.38)	< 0.001^***^	1.28 (1.18 ~ 1.38)	< 0.001^***^
Q3	1.87 (1.74 ~ 2.01)	< 0.001^***^	1.73 (1.61 ~ 1.86)	< 0.001^***^	1.72 (1.60 ~ 1.85)	< 0.001^***^	1.73 (1.60 ~ 1.86)	< 0.001^***^
Q4	3.19 (2.98 ~ 3.41)	< 0.001^***^	2.72 (2.52 ~ 2.93)	< 0.001^***^	2.69 (2.50 ~ 2.90)	< 0.001^***^	2.62 (2.43 ~ 2.82)	< 0.001^***^
P for trend	1.07 (1.06 ~ 1.07)	< 0.001^***^	1.06 (1.05 ~ 1.06)	< 0.001^***^	1.06 (1.05 ~ 1.06)	< 0.001^***^	1.06 (1.05 ~ 1.06)	< 0.001^***^

Model 1: Crude. Model 2: Adjust: Age, Sex, Race, Education, marital status, Wealth, BMI. Model 3: Adjust: Age, Sex, Race, Education, marital status, Wealth, BMI, Drinking status, Smoking status. Model 4: Adjust: Age, Sex, Race, Education, marital status, Wealth, BMI, Drinking status, Smoking status, Physical activity. Significance: ^*^ *p* < 0.05, ^**^p < 0.01, ^***^p < 0.001.

**Figure 2:**
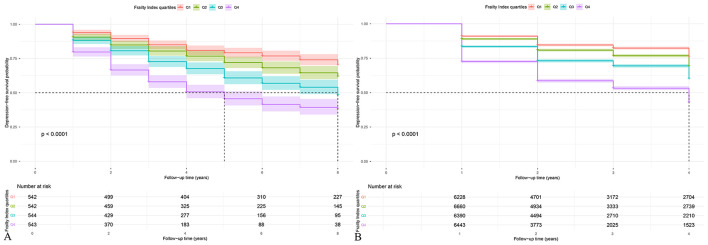
Kaplan–Meier curves for new-onset depression according to frailty index quartiles. **(A)** HRS cohort; **(B)** SHARE cohort.

Kaplan–Meier analyses in HRS and SHARE further supported these findings, showing progressively lower depression-free survival among participants with higher frailty burden over time (Figure 2).

### Competing risk analysis considering death

Fine–Gray competing-risk analyses yielded findings highly consistent with the primary Cox models. Even after accounting for death as a competing event, higher FI remained a significant independent predictor of new-onset depression across all cohorts. The similarity between competing-risk and primary analyses suggests that the frailty–depression association was robust and not substantially explained by differential mortality (Table 5).

**Table 5:** Fine–Gray competing risk regression analysis of the association between the Frailty Index and new-onset depression, treating death as a competing event.

Exposure variable	Model 1		Model 2		Model 3		Model 4	
	sHR (95% CI)	*P*-value	sHR (95% CI)	*P*-value	sHR (95% CI)	*P*-value	sHR (95% CI)	*P*-value
HRS								
15.6-7.4,-14.3498ptFrailty	1.04 (1.03 ~ 1.05)	< 0.001^***^	1.03 (1.03 ~ 1.04)	< 0.001^***^	1.03 (1.03 ~ 1.04)	< 0.001^***^	1.03 (1.03 ~ 1.04)	< 0.001^***^
Frailty group								
Q1	1.00 (reference)	–	1.00 (reference)	–	1.00 (reference)	–	1.00 (reference)	–
Q2	1.37 (1.10 ~ 1.71)	0.005^**^	1.32 (1.05 ~ 1.66)	0.016^*^	1.31 (1.04 ~ 1.64)	0.020^*^	1.31 (1.04 ~ 1.64)	0.020^*^
Q3	1.96 (1.59 ~ 2.42)	< 0.001^***^	1.86 (1.48 ~ 2.33)	< 0.001^***^	1.84 (1.47 ~ 2.31)	< 0.001^***^	1.83 (1.46 ~ 2.30)	< 0.001^***^
Q4	3.05 (2.49 ~ 3.74)	< 0.001^***^	2.72 (2.16 ~ 3.42)	< 0.001^***^	2.66 (2.11 ~ 3.35)	< 0.001^***^	2.62 (2.08 ~ 3.31)	< 0.001^***^
15.6-7.4,-14.3498ptP for trend	1.05 (1.04 ~ 1.06)	< 0.001^***^	1.04 (1.03 ~ 1.05)	< 0.001^***^	1.04 (1.03 ~ 1.05)	< 0.001^***^	1.04 (1.03 ~ 1.05)	< 0.001^***^
ELSA								
15.6-7.4,-14.3498ptFrailty	1.04 (1.04 ~ 1.04)	< 0.001^***^	1.04 (1.04 ~ 1.04)	< 0.001^***^	1.04 (1.04 ~ 1.04)	< 0.001^***^	1.04 (1.03 ~ 1.04)	< 0.001^***^
Frailty group								
Q1	1.00 (reference)	–	1.00 (reference)	–	1.00 (reference)	–	1.00 (reference)	–
Q2	1.93 (1.47 ~ 2.52)	< 0.001^***^	2.01 (1.54 ~ 2.64)	< 0.001^***^	2.02 (1.54 ~ 2.65)	< 0.001^***^	2.03 (1.55 ~ 2.67)	< 0.001^***^
Q3	3.13 (2.44 ~ 4.03)	< 0.001^***^	3.31 (2.56 ~ 4.27)	< 0.001^***^	3.33 (2.57 ~ 4.30)	< 0.001^***^	3.29 (2.54 ~ 4.25)	< 0.001^***^
Q4	7.03 (5.57 ~ 8.87)	< 0.001^***^	7.26 (5.69 ~ 9.27)	< 0.001^***^	7.20 (5.63 ~ 9.23)	< 0.001^***^	6.50 (5.05 ~ 8.35)	< 0.001^***^
15.6-7.4,-14.3498ptP for trend	1.07 (1.06 ~ 1.07)	< 0.001^***^	1.07 (1.06 ~ 1.07)	< 0.001^***^	1.06 (1.06 ~ 1.07)	< 0.001^***^	1.06 (1.05 ~ 1.07)	< 0.001^***^
SHARE								
15.6-7.4,-14.3498ptFrailty	1.04 (1.04 ~ 1.04)	< 0.001^***^	1.03 (1.03 ~ 1.04)	< 0.001^***^	1.03 (1.03 ~ 1.04)	< 0.001^***^	1.03 (1.03 ~ 1.04)	< 0.001^***^
Frailty group								
Q1	1.00 (reference)	–	1.00 (reference)	–	1.00 (reference)	–	1.00 (reference)	–
Q2	1.30 (1.21 ~ 1.40)	< 0.001^***^	1.27 (1.18 ~ 1.37)	< 0.001^***^	1.27 (1.18 ~ 1.36)	< 0.001^***^	1.27 (1.18 ~ 1.37)	< 0.001^***^
Q3	1.81 (1.69 ~ 1.94)	< 0.001^***^	1.69 (1.58 ~ 1.81)	< 0.001^***^	1.68 (1.57 ~ 1.80)	< 0.001^***^	1.69 (1.57 ~ 1.81)	< 0.001^***^
Q4	2.93 (2.75 ~ 3.12)	< 0.001^***^	2.55 (2.38 ~ 2.73)	< 0.001^***^	2.52 (2.35 ~ 2.70)	< 0.001^***^	2.46 (2.29 ~ 2.64)	< 0.001^***^
P for trend	1.06 (1.06 ~ 1.07)	< 0.001^***^	1.05 (1.05 ~ 1.06)	< 0.001^***^	1.05 (1.05 ~ 1.06)	< 0.001^***^	1.05 (1.05 ~ 1.05)	< 0.001^***^

Model 1: Crude. Model 2: Adjust: Age, Sex, Race, Education, marital status, Wealth, BMI. Model 3: Adjust: Age, Sex, Race, Education, marital status, Wealth, BMI, Drinking status, Smoking status. Model 4: Adjust: Age, Sex, Race, Education, marital status, Wealth, BMI, Drinking status, Smoking status, Physical activity. Significance: ^*^p < 0.05, ^**^p < 0.01, ^***^p < 0.001.

### Discrete-time logistic regression analysis

Discrete-time models further confirmed the stability of the observed association across repeated follow-up intervals. Higher FI consistently predicted greater odds of incident depression over time, and participants in the highest FI categories remained at markedly elevated risk. These results closely mirrored the Cox and competing-risk findings, demonstrating strong methodological consistency across analytical approaches (Table 6).

**Table 6:** Discrete-time logistic regression analysis of the association between the Frailty Index and the risk of new-onset depression.

Exposure variable	Model 1		Model 2		Model 3		Model 4	
	OR (95% CI)	*P*-value	OR (95% CI)	*P*-value	OR (95% CI)	*P*-value	OR (95% CI)	*P*-value
HRS								
15.6-7.4,-14.3498ptFrailty	1.05 (1.04 ~ 1.05)	< 0.001^***^	1.04 (1.03 ~ 1.05)	< 0.001^***^	1.04 (1.03 ~ 1.05)	< 0.001^***^	1.04 (1.03 ~ 1.05)	< 0.001^***^
Frailty group								
Q1	1.00 (reference)	–	1.00 (reference)	–	1.00 (reference)	–	1.00 (reference)	–
Q2	1.32 (1.04 ~ 1.67)	0.025^*^	1.26 (0.99 ~ 1.62)	0.066	1.25 (0.98 ~ 1.61)	0.076	1.25 (0.98 ~ 1.60)	0.078
Q3	2.00 (1.59 ~ 2.51)	< 0.001^***^	1.89 (1.48 ~ 2.42)	< 0.001^***^	1.87 (1.47 ~ 2.40)	< 0.001^***^	1.87 (1.46 ~ 2.39)	< 0.001^***^
Q4	3.32 (2.66 ~ 4.15)	< 0.001^***^	2.95 (2.30 ~ 3.79)	< 0.001^***^	2.88 (2.24 ~ 3.71)	< 0.001^***^	2.84 (2.21 ~ 3.66)	< 0.001^***^
15.6-7.4,-14.3498ptP for trend	1.05 (1.04 ~ 1.06)	< 0.001^***^	1.05 (1.04 ~ 1.06)	< 0.001^***^	1.05 (1.04 ~ 1.06)	< 0.001^***^	1.05 (1.04 ~ 1.06)	< 0.001^***^
SHARE								
15.6-7.4,-14.3498ptFrailty	1.05 (1.05 ~ 1.06)	< 0.001^***^	1.05 (1.04 ~ 1.05)	< 0.001^***^	1.05 (1.04 ~ 1.05)	< 0.001^***^	1.05 (1.04 ~ 1.05)	< 0.001^***^
Frailty group								
Q1	1.00 (reference)	–	1.00 (reference)	–	1.00 (reference)	–	1.00 (reference)	–
Q2	1.32 (1.22 ~ 1.44)	< 0.001^***^	1.30 (1.19 ~ 1.41)	< 0.001^***^	1.29 (1.19 ~ 1.41)	< 0.001^***^	1.29 (1.19 ~ 1.41)	< 0.001^***^
Q3	1.92 (1.78 ~ 2.09)	< 0.001^***^	1.80 (1.65 ~ 1.96)	< 0.001^***^	1.79 (1.64 ~ 1.94)	< 0.001^***^	1.79 (1.65 ~ 1.95)	< 0.001^***^
Q4	3.51 (3.26 ~ 3.79)	< 0.001^***^	3.05 (2.80 ~ 3.32)	< 0.001^***^	3.02 (2.77 ~ 3.28)	< 0.001^***^	2.92 (2.68 ~ 3.18)	< 0.001^***^
P for trend	1.07 (1.07 ~ 1.08)	< 0.001^***^	1.07 (1.06 ~ 1.07)	< 0.001^***^	1.06 (1.06 ~ 1.07)	< 0.001^***^	1.06 (1.06 ~ 1.07)	< 0.001^***^

Model 1: Crude. Model 2: Adjust: Age, Sex, Race, Education, marital status, Wealth, BMI. Model 3: Adjust: Age, Sex, Race, Education, marital status, Wealth, BMI, Drinking status, Smoking status. Model 4: Adjust: Age, Sex, Race, Education, marital status, Wealth, BMI, Drinking status, Smoking status, Physical activity. Significance: ^*^p < 0.05, ^***^p < 0.001.

### Subgroup analyses

Subgroup analyses examined potential effect modification by sex, age, and physical activity. The positive association between FI and new-onset depression was observed across all subgroups.

**Figure 3:**
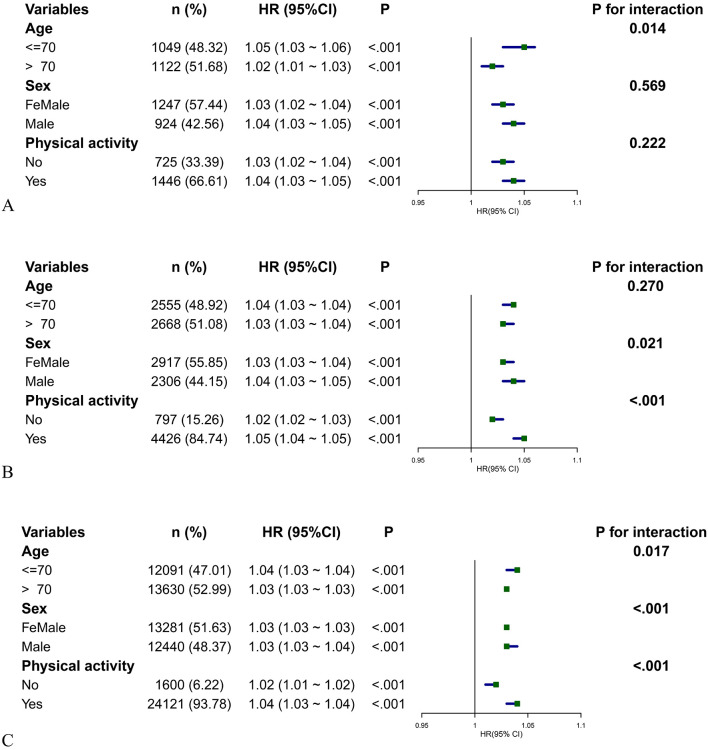
Subgroup analyses of the association between frailty index and new-onset depression. **(A)** HRS cohort; **(B)** ELSA cohort; **(C)** SHARE cohort.

Significant interactions were identified in selected cohorts. In HRS, the association varied by age (*P* for interaction = 0.014). In ELSA, significant interactions were observed for sex (*P* for interaction = 0.021) and physical activity (*P* for interaction < 0.001). In SHARE, interactions were detected for age (*P* for interaction = 0.017), sex (*P* for interaction < 0.001), and physical activity (*P* for interaction < 0.001) (Figure 3).

### Linear and nonlinear associations

**Figure 4:**
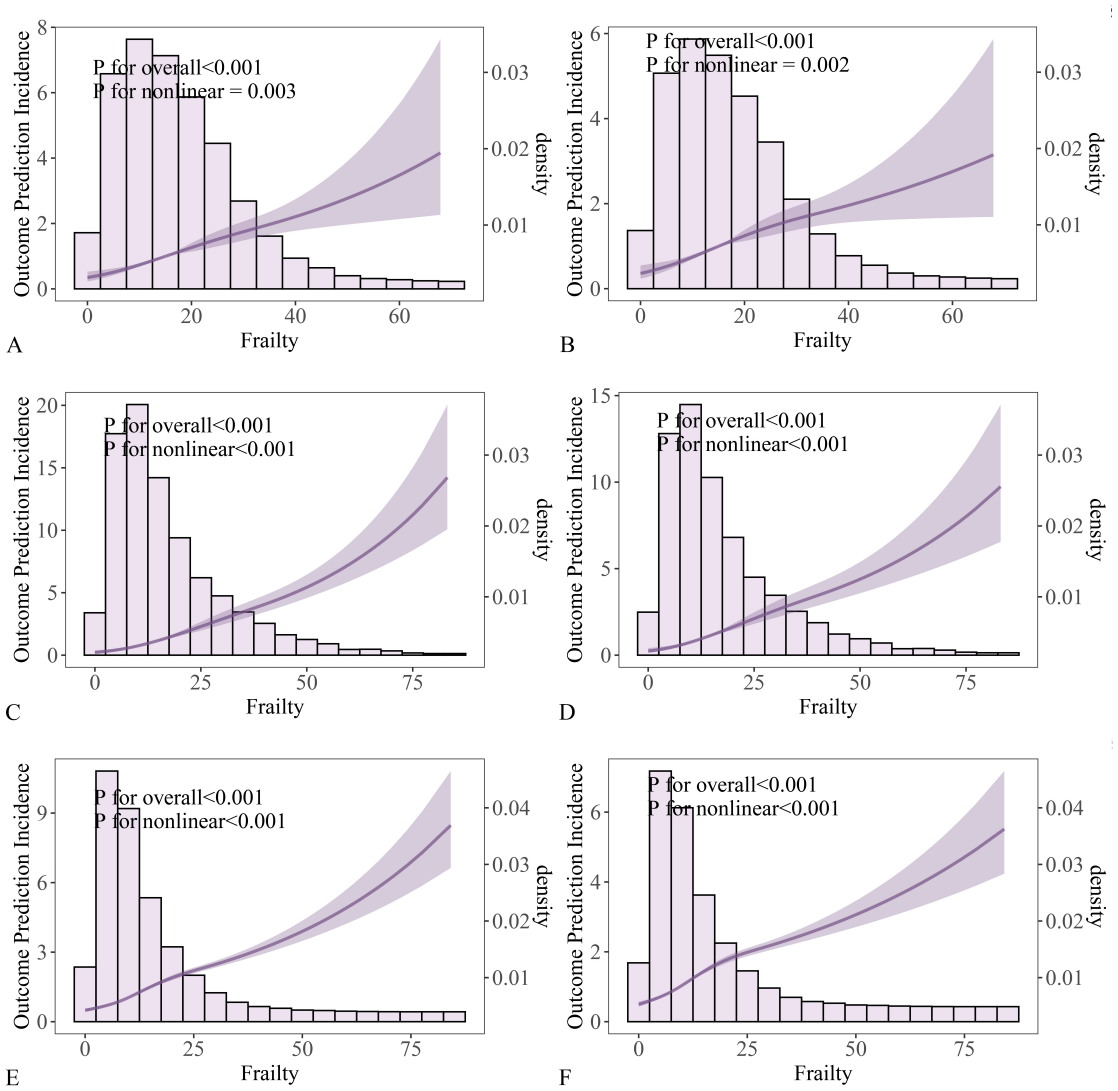
Restricted cubic spline analyses of the association between frailty index and new-onset depression. **(A)** HRS cohort unadjusted model; **(B)** HRS cohort multivariable-adjusted model; **(C)** ELSA cohort unadjusted model; **(D)** ELSA cohort multivariable-adjusted model; **(E)** SHARE cohort unadjusted model; **(F)** SHARE cohort multivariable-adjusted model.

Restricted cubic spline analyses were conducted to evaluate potential nonlinearity. In unadjusted models, significant nonlinear associations between FI and new-onset depression were detected in HRS (Figure 4A), ELSA (Figure 4C), and SHARE (Figure 4E) (*p* for nonlinear < 0.05). These nonlinear patterns persisted after multivariable adjustment (HRS: *p* for nonlinear = 0.002, *p* for overall < 0.001; ELSA: *p* for nonlinear < 0.001, *p* for overall < 0.001; SHARE: *p* for nonlinear < 0.001, *p* for overall < 0.001) (Figures 4B, D, F).

Threshold analyses identified inflection points at 5.92 in HRS, 20.316 in ELSA, and 20.833 in SHARE. On both sides of these thresholds, higher FI values were associated with increased depression risk, although the slope of the association attenuated beyond the inflection points (Table 7).

**Table 7:** Threshold effect analysis of the association between the Frailty Index and new-onset depression using piecewise regression models.

Parameter	HR (95% CI), *P*-value
HRS	
Model 1 Fitting model by standard linear regression	1.037 (1.03–1.044), < 0.001
Model 2 Fitting model by two-piecewise linear regression	
Inflection point	5.92
< 5.92	1.291 (1.149–1.45), < 0.001
>5.92	1.031 (1.024–1.039), < 0.001
P for likelihood ratio test	< 0.001
ELSA	
Model 1 Fitting model by standard linear regression	1.044 (1.039–1.049), < 0.001
Model 2 Fitting model by two-piecewise linear regression	
Inflection point	20.316
< 20.316	1.096 (1.08–1.113), < 0.001
>20.316	1.029 (1.023–1.036), < 0.001
P for likelihood ratio test	< 0.001
SHARE	
Model 1 Fitting model by standard linear regression	1.039 (1.037–1.041), < 0.001
Model 2 Fitting model by two-piecewise linear regression	
Inflection point	20.833
< 20.833	1.064 (1.06–1.069), < 0.001
>20.833	1.013 (1.009–1.018), < 0.001

Model 1: Standard linear regression with CES-D as a continuous predictor. Model 2: Two-piecewise linear regression with CES-D split at the inflection point. Significance: *P* < 0.05 indicates statistical significance.

### Mediation analysis

**Figure 5:**
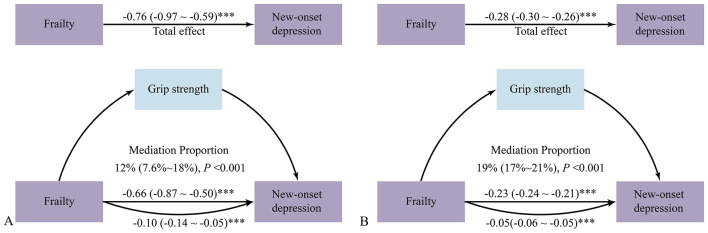
Mediation analysis of muscle strength in the association between frailty index and new-onset depression. **(A)** HRS cohort; **(B)** SHARE cohort. Significance: ^***^p < 0.001.

Figure 5 illustrates the mediating role of grip strength in the association between FI and new-onset depression. Grip strength partially mediated this relationship. The mediated proportion was approximately 12% in HRS and 19% in SHARE. Mediation analysis was not conducted in ELSA because of the limited number of follow-up waves.

## Discussion

This study leveraged data from three nationally representative longitudinal cohorts of older adults—HRS, ELSA, and SHARE—to systematically examine the association between the FI and new-onset depression. Across diverse countries and sociocultural contexts, higher FI values were consistently associated with an increased risk of developing depression. The association remained robust after comprehensive multivariable adjustment and was further confirmed in competing risk and discrete-time modeling frameworks. Dose–response analyses demonstrated a significant nonlinear relationship, with evidence of a threshold effect linking frailty severity to depression risk. Importantly, the predictive value of the FI persisted after accounting for the competing risk of death. Collectively, these findings indicate that frailty represents not merely a marker of physical decline but an independent predictor of subsequent depression. The reproducibility of results across three international cohorts further strengthens their external validity.

Prior studies have consistently reported a close association between frailty and depression, although most available evidence has important methodological limitations. For example, the systematic review and meta-analysis by Soysal et al. demonstrated a strong bidirectional association between frailty and depression in older adults, but the majority of included studies were cross-sectional, thereby limiting temporal inference (18). Similarly, Vaughan et al. highlighted substantial overlap between frailty and depressive symptoms but noted that causal pathways remained uncertain because longitudinal evidence was scarce (19). More recently, Zhou et al. further confirmed that frailty was associated with increased depressive risk, yet heterogeneity in frailty definitions and study design remained a major concern (12). Among prospective studies, Borges et al. reported that frailty predicted subsequent late-life depression in a single-country cohort, while Marconcin et al. extended these findings across European populations but were still constrained by regional frameworks and varying frailty criteria (14, 15). Azizzadeh et al. also demonstrated longitudinal links between depression, anxiety, and frailty, although their focus was primarily on psychological contributors to frailty progression rather than frailty as a predictor of incident depression (17).

Our findings are broadly consistent with these prior reports while substantially extending the literature in several important ways. First, by simultaneously analyzing three nationally representative cohorts from the United States, England, and multiple European countries using harmonized FI methodology, our study provides stronger cross-national external validity than most previous single-country or region-specific investigations. Second, unlike many prior studies, we specifically focused on new-onset depression after excluding baseline depression, thereby strengthening temporal interpretation. Third, the incorporation of competing-risk analyses, nonlinear dose–response assessment, and mediation analyses involving grip strength provides methodological depth beyond much of the existing literature. Collectively, these advances suggest that frailty is not only associated with depression but may represent a robust early-life-course vulnerability marker for incident depressive disorders in aging populations.

Several interconnected biological and behavioral mechanisms may underlie the observed association. Biologically, frailty is frequently accompanied by chronic low-grade inflammation, reflected in elevated interleukin-6 (IL-6) and C-reactive protein (CRP) levels. Persistent inflammation may dysregulate the hypothalamic–pituitary–adrenal axis, leading to cortisol imbalance and impaired neuroplasticity (23). Shared inflammatory pathways may also contribute to neuronal injury in mood-regulating regions, including the hippocampus, thereby facilitating depressive symptomatology (24). Functionally, frailty compromises activities of daily living (ADL) and instrumental activities of daily living (IADL). These impairments can reduce autonomy, restrict social participation, and precipitate role loss, ultimately increasing loneliness and social isolation (25). Such psychosocial stressors may heighten depression risk by undermining self-efficacy and weakening social connectedness (26). Moreover, frailty is characterized by diminished physiological reserve, which increases vulnerability to external stressors. This aligns with the stress–vulnerability model, whereby reduced resilience amplifies the psychological impact of adverse life events and accelerates cognitive decline, thereby elevating depression risk (27). Muscle weakness, commonly assessed by grip strength, may partially mediate this pathway. Through its close association with sarcopenia and functional deterioration, reduced grip strength may link multisystem deficits inherent in frailty to the development of depression (28).

More specifically, our mediation analyses suggest that grip strength may function not merely as a marker of physical decline but as an intermediary biological and functional pathway through which frailty contributes to depression risk. Grip strength is increasingly recognized as a robust surrogate of overall muscle quality, neuromuscular integrity, and sarcopenia severity, all of which are closely linked to aging-related multisystem dysregulation (29, 30). Reduced grip strength may reflect impaired mitochondrial bioenergetics, anabolic resistance, hormonal dysregulation, and chronic inflammatory activation, including elevated IL-6 and TNF-α, which may simultaneously accelerate physical frailty and negatively affect mood regulation through neuroimmune pathways (31, 32). In addition, declining muscular strength often precedes mobility limitation, slower gait speed, fatigue, and loss of independence, thereby reinforcing sedentary behavior, social disengagement, and perceived helplessness—well-established contributors to depressive symptom development (33). Emerging longitudinal evidence further suggests that low grip strength is independently associated with future depressive symptoms and may serve as an early warning biomarker for both physical and psychological vulnerability in older adults (34, 35). Therefore, grip strength may represent a clinically actionable mediator within the frailty–depression pathway, supporting interventions such as resistance training, protein supplementation, and physical rehabilitation not only to improve physical resilience but also to potentially reduce depression risk.

In addition, heterogeneity in frailty assessment—including phenotype-based definitions and varying deficit accumulation indices—has contributed to inconsistent conclusions (36). The present study addressed these limitations by implementing a standardized deficit accumulation FI across three large multinational cohorts. Participants with baseline depression were rigorously excluded to isolate incident cases (17). This design enhanced temporal inference, enabled cross-national validation, and strengthened the robustness of the findings.

Despite substantial differences in healthcare systems, welfare structures, and sociocultural environments, the association between frailty and depression was remarkably consistent across HRS, ELSA, and SHARE (15). The United States operates a comparatively fragmented insurance system, the United Kingdom provides universal healthcare coverage, and SHARE includes multiple European welfare models. Nevertheless, the magnitude and direction of associations were stable across settings (14). In the context of rapid global population aging, these results underscore the generalizability of frailty as a risk factor for depression and suggest that its mental health consequences transcend national and socioeconomic boundaries (37).

From clinical and public health perspectives, the FI may function as a practical screening instrument to identify older adults at heightened risk of depression (38). Early identification could facilitate targeted preventive strategies. Multidisciplinary frailty management—including structured exercise to improve grip strength, nutritional optimization, and psychosocial support—may help reduce depression incidence (39). In aging societies, integrated care models that address frailty comprehensively may mitigate healthcare burden while promoting healthy longevity (40). Given that frailty is at least partially reversible, timely detection is critical for implementing primary prevention initiatives, such as community-based programs designed to enhance physical reserve and foster social engagement (41).

This study possesses several notable strengths. It utilized three large, nationally representative prospective cohorts spanning the United States, England, and multiple European countries, thereby enhancing external validity across diverse healthcare and sociocultural settings. A standardized FI framework was applied across cohorts to improve methodological consistency and cross-national comparability. By excluding participants with baseline depression and incorporating long-term follow-up, the study strengthened temporal inference regarding incident depression. In addition, the use of competing-risk analyses, nonlinear dose–response modeling, and mediation analyses involving grip strength provided methodological rigor beyond much of the prior literature (42).

Several limitations should also be considered. First, depression outcomes were assessed using self-reported symptom scales rather than formal clinical diagnoses, which may introduce misclassification bias and limit diagnostic precision (43). Second, although extensive covariate adjustment was performed, residual confounding from unmeasured factors—such as genetic predisposition, antidepressant use, social support quality, or other psychosocial variables—cannot be entirely excluded (44). Third, despite excluding baseline depression, reverse causality remains possible if subclinical depressive symptoms influenced frailty status prior to formal depression onset (45). Fourth, participant attrition during longitudinal follow-up may have introduced survivor bias, potentially resulting in disproportionate retention of healthier individuals and underestimation of associations (46). Finally, although substantial efforts were made to harmonize variables across HRS, ELSA, and SHARE, differences in measurement frameworks, cultural reporting patterns, and cohort-specific assessment procedures may still have introduced some degree of heterogeneity. Therefore, while the consistency of findings across cohorts strengthens confidence, causal interpretation should remain cautious.

## Conclusion

Among older adults across the United States, the United Kingdom, and Europe, higher frailty index values were consistently associated with an increased risk of new-onset depression, with robust dose–response, nonlinear, and competing-risk evidence. Grip strength partially mediated this relationship, highlighting muscle function as a potential intervention target. Routine frailty and grip strength screening in older adults may facilitate early identification of depression risk, while strength-promoting, nutritional, and psychosocial interventions could help reduce late-life depression burden. Public health policies that prioritize frailty prevention and integrated healthy aging strategies may further improve mental health outcomes in aging populations.

## Data Availability

The raw data supporting the conclusions of this article will be made available by the authors, without undue reservation.
